# Headspace GC–MS volatiles profiling in leaves of 4 *Cymbopogon* species and their vapor-phase antibacterial effects

**DOI:** 10.1038/s41598-026-45553-7

**Published:** 2026-04-17

**Authors:** Mariam O. Wahdan, Fatema R. Saber, Mariam Hassan, Marwa E. Hassan, Mohamed A. Farag

**Affiliations:** 1 Postgraduate Program in Pharmacognosy Department, Faculty of Pharmacy, Cairo University, Kasr El Aini St, Cairo, 11562 Egypt; 2 Pharmacognosy Department, Faculty of Pharmacy, Cairo University, Kasr El Aini St, Cairo, 11562 Egypt; 3 Department of Microbiology and Immunology, Faculty of Pharmacy, Cairo University, Kasr El Aini St, Cairo, 11562 Egypt; 4 Department of Microbiology and Immunology, Faculty of Pharmacy, Galala University, New Galala City, Suez, Egypt; 5 Egyptian Drug Authority, Giza, 12553 Egypt

**Keywords:** *Cymbopogon*, Lemongrass, SPME, Head Space GC-MS, Vapor phase -MIC, Antibacterial, Microbiology, Plant sciences

## Abstract

Aromatic *Cymbopogon* grasses are valued for their culinary and medicinal applications, yet most species remain underexplored compared to *C. citratus.* In this study, the aroma profile of leaves from 4 *Cymbopogon* species (*C. citratus*, *C. nardus*, *C. martini* and *C. procerus*) was characterized using headspace-SPME-GC-MS-based analysis alongside lemongrass essential oil identifying 44 volatiles. *C. citratus*, *C. nardus*, *C. procerus*, and lemongrass oil were rich in aldehydes, predominantly citral isomers, neral (24.6–33.9%) and geranial (38.5–57.1%), while geraniol alcohol (86.6%) characterized *C. martini*. Multivariate data analyses were utilized for species classification but failed to reveal geographical origin. The vapor-phase antibacterial activity of *Cymbopogon* leaves, lemongrass oil, and citral was evaluated against multidrug-resistant ESKAPE pathogens (*Enterococcus faecalis*, *Staphylococcus aureus*, *Klebsiella pneumoniae*, *Acinetobacter baumannii*, *Pseudomonas aeruginosa*, and *Enterobacter cloacae*). Each specimen revealed inhibition of all Gram-positive and Gram-negative bacteria except *E. faecalis*, for which only one *C. citratus* (*ctr3*) and lemongrass oil were effective at vapor-phase minimum inhibitory concentration (VP-MIC) 1.57 mg/mL and 0.1 µg/mL, respectively. Lemongrass oil showed higher activity than citral against *K. pneumoniae* (0.05 vs. 0.1 µg/mL) and *A. baumannii* (0.08 vs. 0.12 µg/mL). The composition of *C. procerus* aroma is revealed for the first time along its antibacterial effects.

**Supplementary Information:**

The online version contains supplementary material available at 10.1038/s41598-026-45553-7.

## Introduction

Humans have exploited herbs since early history^1^, owing to their rich aroma composition as well as their culinary and health-improving properties^2^. Within the grass family, Poaceae (Gramineae), the genus *Cymbopogon* is particularly notable for its richness in essential oils of food and medicinal value^3^. The genus comprises approximately 144 species widely cultivated in tropical and subtropical regions^4^. The name *Cymbopogon* is derived from the Greek words *kymbe* (boat), describing the spathes, and *pogon* (beard), referring to the inflorescence spikes^5^.

In folk medicine, *Cymbopogon* species have been traditionally used to treat infectious diseases, influenza, fever, gastrointestinal disorders, toothaches, headaches, fatigue, mental illness, and skin inflammation^6^. Beyond food flavoring, *Cymbopogon* exhibits diverse biological activities, including insect-repellent, anthelmintic, anticancer, antihyperglycemic, antihyperlipidemic, anti-inflammatory, and antibacterial effects^7^. Its rich aroma composition underlies many of these applications, particularly in insect control and the cosmetic and pharmaceutical industries^8^. Major volatile constituents commonly reported in *Cymbopogon* essential oils include citral (neral and geranial), nerol, geraniol, myrcene, limonene, and citronellal, with marked variation among different chemotypes^9^. Among the most commercially important aroma-bearing grasses are *C. citratus* (lemongrass), *C. nardus* (citronella), and *C. martini* (palmarosa), which are extensively cultivated for essential oil production and widely used in perfumery, cosmetics, food applications, household products, and as insect repellents^10^. Despite extensive investigation of the genus, most studies have focused on *C. citratus*, with comparatively limited attention given to other species and chemotypes^11^.

Solid-phase microextraction (SPME) is a simple, sensitive, non-destructive, and solvent-free technique for volatile profiling^12^, and when coupled with gas chromatography–mass spectrometry (GC–MS) it enables detailed characterization of aroma composition^13^. In parallel, multidrug-resistant (MDR) organisms—commonly referred to as “ESKAPE” pathogens—are responsible for the majority of nosocomial infections due to their ability to evade the biocidal effects of multiple antimicrobial agents^14^. These pathogens contribute substantially to global morbidity, mortality, and healthcare costs^15^ and include *Enterococcus faecalis*, *Staphylococcus aureus*, *Klebsiella pneumoniae*, *Acinetobacter baumannii*, *Pseudomonas aeruginosa*, and *Enterobacter* species^15^, all of which have been designated as critical priority pathogens by the World Health Organization^16^.

Essential oil–bearing plants represent a promising source of antimicrobial agents^17^. In addition to their intrinsic antibacterial activity, essential oils may act synergistically with conventional antibiotics, offering potential strategies to combat MDR pathogens^18^. Their multicomponent nature provides an advantage over single-compound antibiotics by simultaneously targeting multiple bacterial pathways, thereby reducing the likelihood of resistance development^19^. While essential oils have been extensively studied for antimicrobial activity in the liquid phase, comparatively less emphasis has been placed on their vapor-phase effects^20^. Importantly, vapor-phase application has been shown to offer several advantages, including enhanced antimicrobial efficacy, lower effective concentrations, and ease of application^21,22^.

Although the vapor-phase antibacterial activity of *Cymbopogon* essential oils has been reported previously^17,21,23–25^, these studies vary considerably in scope and methodology. Prior investigations have primarily focused on distilled essential oils, formulated vapor blends, or isolated compounds, with chemical characterization generally performed on the oils themselves rather than on volatiles emitted directly from fresh plant tissues. Collectively, these findings demonstrated the antimicrobial potential of *Cymbopogon* essential oils in the vapor phase, while highlighting that the vapor-phase antibacterial activity of volatiles emitted directly from intact *Cymbopogon* leaves has not been systematically evaluated. Moreover, *Cymbopogon procerus* remains a poorly investigated species, and to the best of our knowledge, neither its volatile profile nor its antibacterial activity—particularly in the vapor phase—has been previously reported.

While HS-SPME–GC–MS has been previously applied to certain *Cymbopogon* species-most often focusing on essential oils or individual species^26,27^-the present study applies a comparative headspace approach to volatiles emitted directly from intact leaves across multiple *Cymbopogon* species under identical experimental conditions.

To address these gaps, the present study aimed to comparatively characterize the volatile profiles of *Cymbopogon* leaves using headspace SPME–GC–MS among 4 *Cymbopogon* species. The analysis included three *C. citratus* leaf samples from two geographical locations, together with leaves of three additional *Cymbopogon* species, all analyzed under identical experimental conditions. A commercial lemongrass essential oil was included as a reference material to enable comparison between volatiles emitted directly from leaves and those obtained using steam distillation, resulting in a total of 7 samples (Table 1). In parallel, the vapor-phase antibacterial activity of volatiles emitted from *Cymbopogon* leaves, lemongrass essential oil, and the major compound citral were evaluated against a panel of 9 ESKAPE pathogens, and the microbiological responses were comparatively assessed.

## Materials and methods

### Plant material, fibers and chemicals

Plant material (leaves of four *Cymbopogon* species: *C. citratus*, *C. nardus*, *C. martini*, and *C. procerus*, represented by six specimens) was obtained from official botanical garden herbarium sources as listed in Table 1, with voucher numbers provided for all samples. All experimental procedures were conducted in accordance with relevant laws and institutional guidelines, including appropriate permissions for the use of plant specimens obtained from herbaria. Authentication was accomplished by the following specialists listed in each Herbarium. Prof. Dr. Peter Poschlod at Botanical Garden, Regensburg, Germany, Dr. Heike Elisabeth Schwarzer at Ecological Botanical Garden, Bayreuth, Germany, Prof. Dr. Stefan Porembski at Botanical Garden, Rostock, Germany, and Prof. Dr. Dietmar Quandt at Botanical Garden, Bonn, Germany.

A commercial lemongrass essential oil was purchased from Harraz Company (Cairo, Egypt) and included as a reference sample for comparative profiling (Table 1). All materials were stored at −20 °C in airtight containers until analysis. Headspace SPME was performed using divinylbenzene-carboxen-polydimethylsiloxane (DVB/CAR/PDMS)^28^ that were obtained from Supelco (Bellefonte, PA, USA). All chemicals and analytical-grade standards, including citral (used as an authentic reference standard) and (Z)−3-hexenyl acetate (used as an internal standard), were purchased from Sigma-Aldrich (St. Louis, MO, USA).

**Table 1:** List of Cymbopogon samples under investigation.

Sample	Sample Code	Origin	Voucher number
*Cymbopogon citratus* (DC.) Stapf	*ctr1*	Botanical Garden, Regensburg, Germany	Akz.: 2251
*Cymbopogon citratus* (DC.) Stapf	*ctr2*	Ecological Botanical Garden, Bayreuth, Germany	Akz.: 971,893, IPEN: XX-0-BAYRT-971,893
*Cymbopogon citratus* (DC.) Stapf	*ctr3*	Ecological Botanical Garden, Bayreuth, Germany	Akz.: 021163
*Cymbopogon nardus* (L.) Rendle	*nrd*	Botanical Garden, Rostock, Germany	Akz. 2004-G-67
*Cymbopogon martini* (Roxb.) J.F.Watson	*mrt*	Botanical Garden, Bonn, Germany	XX-0-BONN-19,494
*Cymbopogon procerus* (R.Br.) Domin	*prc*	Botanical Garden, Bonn, Germany	XX-0-BONN-26,667

### Headspace analysis of Cymbopogon volatiles

The GC-MS analysis of volatiles using headspace solid-phase microextraction (HS-SPME) was performed following a previously described method^29^ with minor modifications. Fresh leaves (30 mg) were placed in 1.5 mL screw-capped SPME vials and spiked with (Z)−3-hexenyl acetate (3 µg/vial), dissolved in water, as an internal standard for quality control purposes. The SPME fiber was manually introduced into each vial containing the sample and incubated in an oven at 50 °C for 30 min. The fiber was then withdrawn into the needle and injected into the GC-MS injection port.

*Cymbopogon* samples were analyzed using a Shimadzu GC-17 A gas chromatograph equipped with a DB-5 capillary column (30 m × 0.25 mm i.d. × 0.25 μm film thickness; Supelco), coupled to a Shimadzu QP5050A mass spectrometer. The interface and injector temperatures were set at 220 °C. The oven temperature program was as follows: 40 °C for 3 min, increased to 180 °C at 12 °C/min and held for 5 min. Helium was used as the carrier gas at a flow rate of 0.9 mL/min with a split ratio of 1:50.

The SPME fiber was cleaned before each run by conditioning in the injection port at 220 °C for 2 min. The quadrupole mass spectrometer was operated in electron ionization (EI) mode at 70 eV, with a scan range of 40–500 *m/z*. Three biological replicates were analyzed for each leaf sample.

The commercial lemongrass essential oil was included as a reference material, diluted (3 µL in 1 mL chloroform), and analyzed once using the same GC-MS conditions. Blank injections were performed throughout the analysis. The initial 5 min of each run was excluded as solvent delay.

Within each sample, the relative percentage of each compound was calculated by dividing its peak area by the total peak area of all identified compounds and multiplied by 100. It should be noted that HS-SPME provides semi-quantitative data; therefore, the results are intended for relative comparison under identical experimental conditions.

### Identification of volatiles

Volatile compounds were identified by comparison of their retention indices (RI) relative to a homologous series of *n*-alkanes (C6-C20), mass spectral matching with NIST and Wiley libraries, and confirmation using an authentic reference standard whenever available. Prior to spectral matching, peak deconvolution was performed using AMDIS software (www.amdis.net).

### Screening of antibacterial activity

#### Bacterial strains

A panel of Gram-positive and Gram-negative multidrug-resistant bacterial pathogens was used to evaluate vapor-phase antibacterial activity. The tested strains belong to the ESKAPE group, which includes major causative agents of nosocomial infections^14,30^. Nine standard bacterial strains were tested: *Enterococcus faecalis* ATCC19433, *Staphylococcus aureus* Newman, Methicillin-resistant *Staphylococcus aureus* (MRSA USA300), *Klebsiella pneumoniae* ATCC13883, *Acinetobacter baumannii* AB5075, *Pseudomonas aeruginosa* PAO1, *Enterobacter cloacae*, *Escherichia coli* ATCC8739 and *Salmonella typhi* ATCC35664^31–34^.

#### Vapor-phase minimum inhibitory concentration (VP-MIC)

The vapor-phase antibacterial activity of the tested leaf samples, essential oil and citral was evaluated by determining the vapor-phase minimum inhibitory concentration (VP-MIC) following previously reported methods^21,30,35^.

Briefly, 15 mL of sterile Mueller-Hinton agar (MHA) were pipetted into glass petri dishes (10 cm diameter). After solidification, the agar surface was inoculated by spotting 10 µL of each bacterial suspension (10^6^ CFU/mL). In each Petri dish, all nine microorganisms were tested simultaneously by spotting each bacterial inoculum side by side, as shown in **Supplementary Fig. ****S1****.**

A defined weight of the tested leaf material, essential oil or citral was then placed on the inner surface of the dish cover. The plates were then inverted so that the inoculated agar faced upward and the cover containing the tested sample faced downward. The Petri dishes were immediately sealed with parafilm, incubated at 37 °C, and inspected after 24 h. Control plates were prepared under identical conditions, with the covers left empty and without adding any tested sample.

For *Cymbopogon* leaf samples, multiple plates were prepared at different concentrations (2.4, 1.2, and 0.6 mg/mL), whereas for lemongrass essential oil and citral, concentrations of 0.12, 0.06, and 0.03 µg/mL were tested.

The vapor-phase concentration of each tested sample was calculated by dividing the weight (mg or µg) of the leaf, essential oil, or citral (placed on the cover of the Petri dish) by the volume (mL) of the airspace inside the dish. After incubation, bacterial growth on sample and control plates was compared visually, and VP-MIC values were determined accordingly. The VP-MIC was defined as the lowest concentration of the tested leaf, essential oil, or citral that caused apparent inhibition of visible bacterial growth compared to the control.

The VP-MIC assays were performed in triplicate using three independent experimental replicates, each conducted as a separate experiment at different times following an identical standardized procedure, and VP-MIC values are reported as mean ± SD (standard deviation).

### Statistical analysis

GC-MS analyses were performed in triplicate as technical replicates for each leaf sample to assess analytical reproducibility, and data are presented as mean ± standard deviation (SD).

Vapor-phase minimum inhibitory concentration (VP-MIC) data are presented as mean ± standard deviation (SD) of three independent biological replicates.

All experimental data were analyzed using GraphPad Prism software (version 9.0.0). Statistical significance was determined using Two Way ANOVA followed by Tukey’s and Šídák’s post-hoc test for multiple comparisons. A p-value of < 0.05 was considered statistically significant.

Multivariate data analysis was performed on MS abundance tables derived from GC-MS using SIMCA-P 13.0 (Umetrics, Sweden). To identify key markers, we employed PCA, HCA, and OPLS-DA modeling, with variables subjected to mean-centering and Pareto scaling. Potential biomarkers were extracted from S-plots based on covariance (p) and correlation (pcor), while model validation was evaluated through R2 and Q2 diagnostic indices alongside permutation testing.

## Results and Discussion

The main goal of this study was to comparatively characterize volatile profiles among selected *Cymbopogon* species and *C. citratus* samples from different geographical origins, and to further evaluate their vapor-phase antibacterial activity.

### Aroma profile of Cymbopogon

Headspace SPME coupled with GC-MS analysis enabled the identification of 44 volatile compounds (**Supplementary Fig. ****S1** shows representative GC-MS chromatograms of the analysed samples) including aldehydes (9), ketones (7), alcohols (5), oxides/ethers (7), esters/acids (4), monoterpene hydrocarbons (2), sesquiterpene hydrocarbons (8) and aliphatic hydrocarbons (2) (**Table 2**). Aldehydes predominated in *C. citratus* samples (75.73–89.09%), *C. nardus* (86.15%), *C. procerus* (90.36%) and lemongrass essential oil (76.13%), whereas alcohols represented the major class in *C. martini* (90.24%).

These findings are consistent with previous reports describing *C. citratus* and lemongrass essential oil as aldehyde-rich, primarily due to citral isomers (neral and geranial) and *C. martini* as alcohol-rich, dominated by geraniol^7,26,36–40^. In contrast, the volatile profile of *C. nardus* differed from several previous reports in which citronellal, geraniol, nerol and citronellol were reported as major constituents^7,8,39^. Such variations may reflect differences in geographical origin, plant age, seasonal factors and harvesting or processing conditions^8,41^.

### Aldehydes

Aldehydes therefore represented the predominant volatile class in *C. citratus*,* C. nardus*,* C. procerus*, and lemongrass essential oil (75.73–90.36%), whereas they accounted for only 0.93% in *C. martini*. Geranial and neral, the two isomeric forms of citral, were the predominant aldehydes across these taxa. Aldehydes have been reported to exhibit strong antibacterial activity, primarily through interactions with bacterial cell membrane^42^. Geranial (trans-citral) and neral (cis-citral) are aliphatic monoterpene aldehyde isomers that, together, constitute citral, the odor key marker to which *Cymbopogon’*s distinct lemon-like aroma is attributed^42^. Geranial, with a stronger, more pungent citrus fragrance^43^ and more potent effect^44,45^, and neral, of a sweeter, less intense scent^43^, are contributing to citral’s versatile antimicrobial effects^46^. Geranial/neral ratio was relatively consistent among the examined taxa with geranial at ca. 2 fold higher than neral as such: 56.46%, 30.53% of *ctr*1 (*C. citratus*), 56.28%, 27.23% of *ctr*2 (*C. citratus*), 45.62%, 24.89% of *ctr*3 (*C. citratus*), 57%, 24.63% of *nrd* (*C. nardus*), and 52.27%, 27.21% of *prc* (*C. procerus*), whereas in lemongrass essential oil their levels were more comparable at 38.46%, 33.93%, compared to 0.12%, 0.26% of *mrt* (*C. martini*).

Cinnamaldehyde, an aromatic aldehyde that imparts cinnamon its scent^42^, was the second most abundant aldehyde in all leaf samples, though not detected in lemongrass essential oil. Its content showed marked variation, ranging from 0.31% in *C. martini* to 9.3% in *C. procerus*, suggesting a potential and significant (at *p* < 0.05, Table S1) contribution to the distinctive aroma of *C. procerus*. Compared with leaf aroma, lemongrass essential oil exhibited higher levels of isoneral and isogeranial as the second most dominant aldehydes (0.95% and 1.97%), occurring at approximately three-to fourfold higher concentrations than in leaf specimens Table 2.

**Table 2:** Relative percentage of volatiles in *Cymbopogon* aromas as analyzed by GC–MS (mean ± SD, *n* = 3 for leaf samples). The essential oil was analyzed (*n* = 1). For codes, refer to Table 1.

**no.**	**Name**		**Rt(min)**	**RI**	***ctr1***	***ctr2***	***ctr3***	***nrd***	***mrt***	***prc***	**Essential oil**
**1**	Sulcatone		5.56	934	-	-	-	-	-	0.37 ± 0.14	-
**2**	β-Myrcene		5.58	936	3.73 ± 0.02	2.44 ± 3.6	2.65 ± 2.04	1.61 ± 2.36	0.16 ± 0.14	-	12.34
**3**	α-Phellandrene		5.82	948	-	-	-	-	-	-	0.34
**4**	(Z)-Myroxide		7.23	1032	0.27 ± 0.02	0.3 ± 0.31	0.2 ± 0.18	0.22 ± 0.24	-	-	0.57
**5**	Linalool		7.38	1042	0.37 ± 0.02	0.44 ± 0.21	0.68 ± 0.14	0.37 ± 0.23	2.5 ± 1.1	0.3 ± 0.12	1.4
**6**	2-Undecyne		7.92	1077	-	-	-	-	-	-	0.35
**7**	Exo-isocitral		7.98	1081	0.18 ± 0.05	0.09 ± 0.08	0.09 ± 0.08	0.1 ± 0.09	-	0.16 ± 0.02	0.28
**8**	Chrysanthemal		8.07	1087	-	-	-	-	-	-	0.31
**9**	Citronellal		8.12	1090	-	-	-	-	-	-	0.23
**10**	Menthone		8.15	1091	-	-	-	-	0.08 ± 0.07	0.09 ± 0.01	-
**11**	Isomenthone		8.26	1099	-	-	-	-	0.06 ± 0.05	-	-
**12**	Isoneral		8.27	1099	0.33 ± 0.05	0.26 ± 0.25	0.38 ± 0.03	0.24 ± 0.23	-	0.31 ± 0.1	0.95
**13**	Rose furan oxide		8.37	1106	0.21 ± 0.03	0.18 ± 0.17	0.21 ± 0.18	0.1 ± 0.09	-	0.18 ± 0.05	0.04
**14**	Isogeranial		8.52	1116	0.52 ± 0.09	0.37 ± 0.34	0.32 ± 0.28	0.32 ± 0.3	-	0.61 ± 0.13	1.97
**15**	Estragole		8.58	1121	0.3 ± 0.04	0.19 ± 0.18	0.48 ± 0.1	0.46 ± 0.15	0.24 ± 0.08	0.28 ± 0.05	2.29
**16**	4,8-Dimethyl-1,7-nonadien-4-ol		8.92	1144	0.31 ± 0.04	0.21 ± 0.18	0.74 ± 0.17	0.44 ± 0.1	0.09 ± 0.08	0.42 ± 0.05	-
**17**	Neral		9.37	1177	30.53 ± 0.64	27.23 ± 5.57	24.89 ± 7.42	24.63 ± 5.58	0.26 ± 0.25	27.21 ± 3.86	33.93
**18**	Carvone		9.48	1184	-	-	-	-	-	-	traces*
**19**	Geraniol		9.75	1203	-	0.71 ± 0.73	0.48 ± 0.83	0.87 ± 0.8	86.58 ± 0.8	0.65 ± 0.41	2.22
**20**	(E)-Cinnamaldehyde		9.9	1214	0.92 ± 0.18	2.65 ± 1.71	4.03 ± 3.63	3.38 ± 1.58	0.31 ± 0.37	9.3 ± 3.4	-
**21**	Geranial		9.91	1215	56.46 ± 0.64	56.28 ± 4.9	45.62 ± 3.64	57 ± 2.97	0.12 ± 0.11	52.27 ± 0.75	38.46
**22**	Anethole		9.96	1219	0.73 ± 0.05	0.58 ± 0.17	0.14 ± 0.25	1.24 ± 0.64	0.47 ± 0.6	1.3 ± 0.06	-
**23**	2-Undecanone		9.97	1220	0.6 ± 0.05	1.76 ± 0.41	7.35 ± 0.68	-	-	-	0.22
**24**	Safrole		9.98	1221	-	-	-	1.98 ± 1.21	1.65 ± 0.56	0.8 ± 0.12	-
**25**	Geranyl formate		10.12	1232	0.6 ± 0.02	0.64 ± 0.19	1.5 ± 0.7	0.34 ± 0.39	0.6 ± 0.34	-	traces*
**26**	Geranyl acetate		10.97	1296	1.8 ± 0.18	1.3 ± 0.98	1.02 ± 0.93	1.37 ± 0.48	1.37 ± 0.78	0.71 ± 0.29	2.67
**27**	Geranic acid		11.04	1301	0.37 ± 0.08	1.1 ± 0.85	2.06 ± 2.23	1.3 ± 0.83	-	0.92 ± 0.55	-
**28**	4-Methyltridecane		11.15	1310	0.2 ± 0.09	0.26 ± 0.16	0.92 ± 0.25	0.98 ± 0.93	0.32 ± 0.04	0.71 ± 0.07	-
**29**	Methyl Eugenol		11.27	1320	-	-	-	-	-	-	0.33
**30**	(E)-Caryophyllene		11.43	1333	0.14 ± 0.02	0.18 ± 0.12	0.21 ± 0.22	0.51 ± 0.65	1.46 ± 0.27	0.32 ± 0.07	0.34
**31**	2-Ethylidene-6-methyl-3,5-heptadienal		11.51	1340	0.15 ± 0	0.38 ± 0.24	0.4 ± 0.37	0.48 ± 0.27	0.24 ± 0.01	0.5 ± 0.11	-
**32**	(E)-α-Bergamotene		11.55	1343	0.35 ± 0.02	0.42 ± 0.15	0.63 ± 0.61	0.51 ± 0.18	-	-	0.32
**33**	(E)-β-Famesene		11.74	1359	0.42 ± 0.01	0.68 ± 0.38	0.26 ± 0.25	0.71 ± 0.62	-	-	0.04
**34**	Humulene		11.82	1365	-	-	-	-	0.29 ± 0.26	-	0.11
**35**	α-Curcumene		12.07	1386	0.07 ± 0.03	0.04 ± 0.05	0.14 ± 0.18	0.07 ± 0.07	0.06 ± 0.0	0.16 ± 0.05	-
**36**	(Z, E)-α-Farnesene		12.16	1393	0.24 ± 0.04	0.19 ± 0.14	0.36 ± 0.44	0.4 ± 0.21	0.07 ± 0.07	-	0.19
**37**	2-Tridecanone		12.23	1399	0.2 ± 0.01	1.12 ± 0.68	3.87 ± 3.51	0.37 ± 0.17	0.05 ± 0.04	-	0.1
**38**	γ-Cadinene		12.45	1416	-	-	-	-	-	1.27 ± 0.4	-
**39**	(Z)-Calamenene		12.53	1423	-	-	-	-	-	0.16 ± 0.03	-
**40**	Nerolidol		12.94	1457	-	-	-	-	0.17 ± 0.09	-	-
**41**	Caryophyllene oxide		13.22	1480	-	-	-	-	0.53 ± 0.35	1 ± 0.4	-
**42**	2-Pentadecanone		14.27	1555	-	-	0.37 ± 0.49	-	-	-	-
**43**	(Z, E)-Farnesol		14.5	1572	-	-	-	-	0.9 ± 0.75	-	-
**44**	Geranyl hexanoate		14.76	1590	-	-	-	-	1.42 ± 0.96	-	-
	**Total**				100	100	100	100	100	100	100
	Total Aldehydes				89.09	87.26	75.73	86.15	0.93	90.36	76.13
	Total Alcohols				0.68	1.36	1.9	1.68	90.24	1.37	3.62
	Total Ketones				0.8	2.88	11.59	0.37	0.19	0.46	0.32
	Total Oxides/Ethers				1.51	1.25	1.03	4	2.89	3.56	3.23
	Total Esters/Acids				2.77	3.04	4.58	3.01	3.39	1.63	2.67
	Total Monoterpene Hydrocarbons				3.73	2.44	2.65	1.61	0.16	0	12.68
	Total Sesquiterpene Hydrocarbons				1.22	1.51	1.6	2.2	1.88	1.91	1
	Total Aliphatic Hydrocarbons				0.2	0.26	0.92	0.98	0.32	0.71	0.35

* ”traces” indicates compounds detected below the reliable quantification limit.

Compounds were identified based on retention indices relative to n-alkanes (C6-C20), mass spectral matching with NIST and Wiley libraries, and comparison with an authentic reference standard for citral.

### Ketones

Ketones represented 0.19–11.59% of the total volatile constituents. Previous studies have reported that ketones, together with other functional groups, may contribute to the overall antimicrobial potential of essential oils through synergistic interactions^35^.

Sulcatone in *C. procerus*, isomenthone in *C. martini*, and menthone in both *C. martini* and *C. procerus* were detected at low levels ranging from 0.06% to 0.37%. In contrast, *C. citratus* samples were characterized by higher ketone contents, accounting for 0.8–11.59% of total volatiles.

Notably, 2-undecanone, a methyl ketone known for its anti-inflammatory, antifungal, and vapor-biopesticide activities^47–49^, was a major ketone constituent in *C. citratus* leaves and lemongrass essential oil, reaching its highest level in *ctr3* (7.35%). In addition, 2-tridecanone, identified in all samples except *C. procerus*, was predominant in *ctr3* (3.87%), while 2-pentadecanone was detected exclusively in *ctr3* (0.37%). These compounds belong to the methyl ketone class^50^.

Owing to their strong odor, ketones contribute to natural insect-repellent and insecticidal properties, and play an important role in the flavor and fragrance industry^48^. These properties may indirectly support air sanitation and food protection, highlighting the potential applications of *Cymbopogon* volatiles in vapor-phase antimicrobial and preservation systems.

### Alcohols

Alcohols represented the major volatile class in *C. martini* (90.24% of total volatiles), compared with 0.68%, 1.36%, 1.90%, 1.68%, 1.37%, and 3.62% in *ctr1*,* ctr2*,* ctr3*,* nrd*,* prc*, and lemongrass essential oil samples, respectively.

Geraniol, the biosynthetic precursor of geranial, was detected as the dominant constituent in *C. martini* (86.58%), and as a rose-scented acyclic monoterpene alcohol, is responsible for its characteristic pleasant aroma^51,52^. This finding suggests enhanced alcohol biosynthesis of this taxon. In contrast, geraniol occurred only at trace levels in *C. citratus*, *C. nardus*, *C. procerus*, and lemongrass essential oil samples. In addition to its diverse biological activities, geraniol exhibits antibacterial, antifungal, insecticidal, and insect-repellent activities^53^.

Linalool, another acyclic monoterpene alcohol with a floral scent^54^, was detected in all *Cymbopogon* samples with highest level observed in *C. martini* (2.5%). Linalool is widely used as a flavoring, fragrance and preservative agent in foods and cosmetics due to its antimicrobial properties^55^.

Furthermore, nerolidol and (Z, E)-farnesol were detected only in *C. martini* at 0.17% and 0.9%, respectively.

### Oxides/ethers

Oxides and ethers accounted for 1.03–4.00% of the total volatile composition of *Cymbopogon* samples and are reported to contribute to antioxidant and antimicrobial activities^56^.

Oxides ranged from 0.32% to 1.18%, with rose furan oxide and (Z)-myroxide detected in *C. citratus* and *C. nardus* at comparable levels. Lemongrass essential oil exhibited the highest content of (Z)-myroxide (0.57%) and the lowest level of rose furan oxide (0.04%). Caryophyllene oxide was detected exclusively in *C. martini* (0.53%) and *C. procerus* (1.00%).

Ethers were detected at relatively low levels (0.62–3.68%), with the highest abundance observed in *C. nardus* (3.68%) versus lowest in *ctr3* (*C. citratus*, 0.62%). The major ether constituents included estragole, anethole, and safrole. Safrole was detected in *C. nardus* (1.98%), *C. martini* (1.65%), and *C. procerus* (0.80%), but was absent in *C. citratus* and lemongrass essential oil. Further, trace amounts of methyl eugenol were detected in lemongrass essential oil (0.33%). Estragole, safrole, and methyl eugenol are common flavor constituents in several herbs and spices but are currently subject to regulatory limitations due to reported genotoxic and carcinogenic effects^57–59^. However, these restrictions mainly concern their use as isolated food additives and do not necessarily apply to their natural occurrence in plant-derived materials^60^. Anethole was detected in most samples (0.14–1.30%) and is widely utilized in food, cosmetic, perfumery, and pharmaceutical industries owing to its characteristic anise-like aroma and reported biological activities^56^.

### Esters & Acids

Esters and acids represented minor yet biologically relevant fractions of the volatile profiles. Esters, which are known to exhibit soothing and antimicrobial properties^61^, were mainly represented by geranyl acetate, detected in all *Cymbopogon* taxa and ranging from 0.71% in *C. procerus* to 2.67% in lemongrass essential oil.

Organic acids were represented exclusively by geranic acid (0.37–2.06%) which was detected in all taxa except *C. martini* and lemongrass essential oil. Geranic acid, a derivative of geraniol and geranial^62^, has been reported to contribute to food flavor enhancement^63^ and preservation through inhibition of tyrosinase-mediated enzymatic oxidation, thereby limiting browning and indirectly supporting antioxidant protection^64^. In addition, its reported antifungal activity^63^ may further enhance food stability and contribute to the overall antimicrobial potential of *Cymbopogon* volatiles.

Within the complex volatile matrix, these minor constituents may play synergistic roles in reinforcing the biological activity of *Cymbopogon* aroma and supporting its potential applications in food preservation.

### Monoterpene and Sesquiterpene Hydrocarbons

Monoterpene hydrocarbons ranged from 0.00% to 12.68%, with the highest levels detected in lemongrass essential oil, mainly due to β-myrcene, which accounted for 12.34% of the total volatiles. Except for *C. procerus* (*prc*), monoterpene hydrocarbons in *Cymbopogon* leaves ranged from 0.16% in *C. martini* to 3.73% in *C. citratus* (ctr1). Notably, it showed significantly higher levels in *ctr1* and *ctr3* in comparison with *prc* and *mrt* leaves. β-Myrcene has been reported to exhibit antioxidant, antibacterial^9^, antifungal, and antimalarial activities^65^. In addition, α-phellandrene was detected in lemongrass essential oil at 0.34%. Notably, monoterpene hydrocarbons were not detected in *C. procerus*.

Sesquiterpene hydrocarbons were present at relatively low levels, accounting for 1.00% to 2.20% of the total volatiles. (E)-Caryophyllene was identified in all specimens at concentrations ranging from 0.14% to 1.46%. This bicyclic sesquiterpene imparts a characteristic woody aroma^66^ and exhibits antimicrobial activity against both Gram-positive and Gram-negative bacteria^67^. Other less abundant sesquiterpenes, including α-curcumene and β-caryophyllene, were detected in all *Cymbopogon* leaf samples but were absent in lemongrass essential oil. Furthermore, γ-cadinene (1.27%) and (Z)-calamenene (0.16%) were detected only in *C. procerus* (*prc*).

### Aliphatic Hydrocarbons

Aliphatic hydrocarbons were detected only at trace levels and were limited to two compounds: 4-methyltridecane (0.20–0.98%) in all *Cymbopogon* leaf samples and 2-undecyne (0.35%) exclusively in lemongrass essential oil.

### Multivariate data analysis of Cymbopogon taxa aroma profiles

Multivariate analyses were applied as exploratory tools to visualize similarities and differences among the volatile profiles of the 7 analysed samples, including three *C. citratus* leaf samples from two geographical locations, leaves of three additional *Cymbopogon* species, and a commercial lemongrass essential oil.

**Fig. 1:**
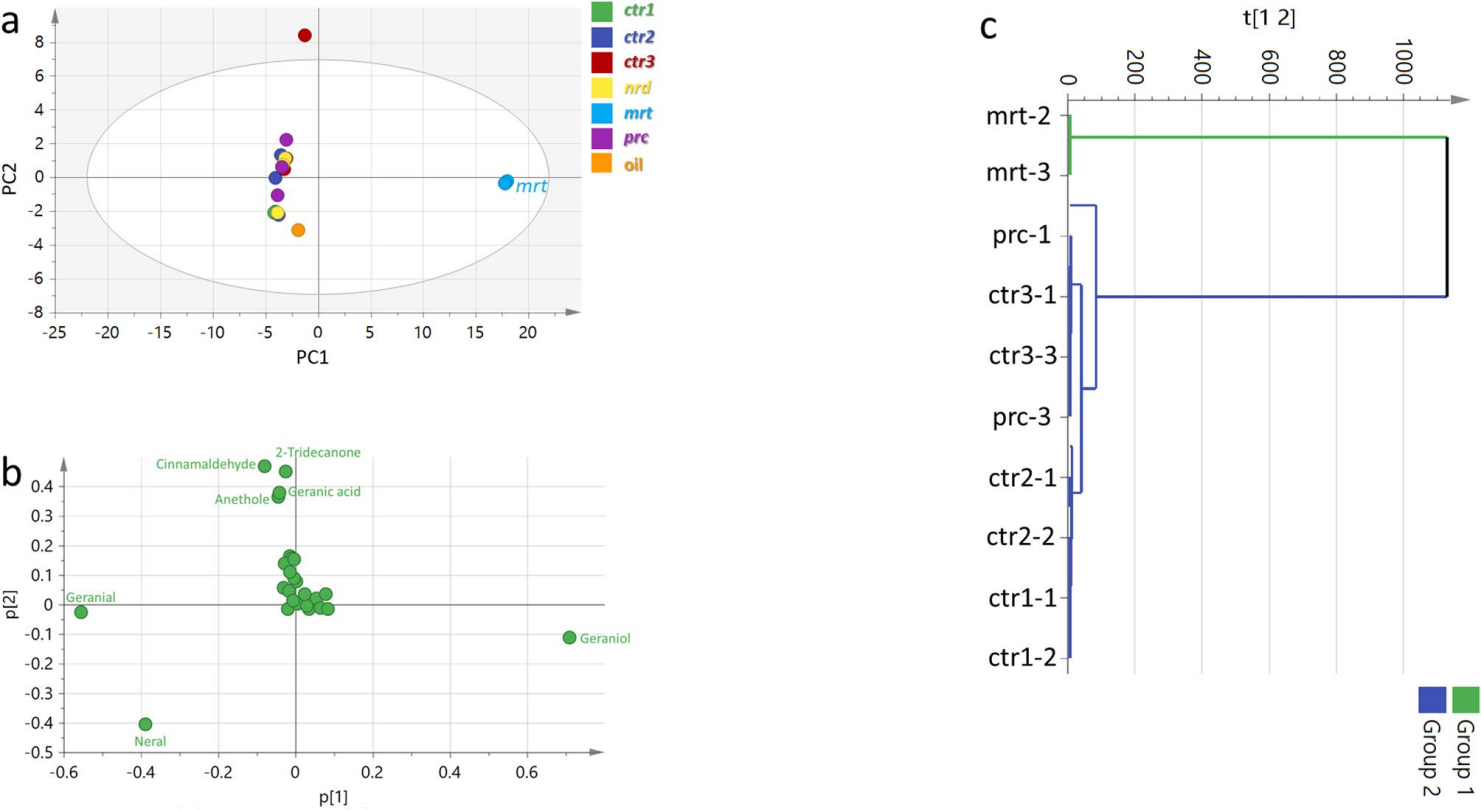
Unsupervised multivariate data analysis of the 7 *Cymbopogon* samples resulting from modelling of volatiles dataset analysed via HS-SPME-GC-MS (*n* = 3). (**a**) PCA score plot of PC1 versus PC2 scores. (**b**) Loading plot for PC1 and PC2, providing mass peaks and their assignments. (**c**) HCA plot. The sample clusters are placed in two-dimensional space at the distinct locations defined by two vectors of principal component PC1 = 78.78% and PC2 = 7.89%. *ctr1*,* ctr2* and *ctr3*,* C. citratus* leaves from 2 origins; *nrd*,* C. nardus* leaves; *mrt*,* C. martini* leaves; *prc*, *C. procerus* leaves; oil, Lemongrass essential oil.

Principal component analysis (PCA) was initially applied to the complete GC-MS dataset. The PCA score plot (Fig. 1a) showed a clear separation of *C. martini* from the other samples along PC1, with PC1 and PC2 explaining 86.67% of the total variance. Examination of the corresponding loading plot indicated that this separation was primarily associated with the higher contribution of geraniol in *C. martini* (Fig. 1b). Hierarchical cluster analysis (HCA) produced a comparable grouping pattern, separating *C. martini* from the remaining samples (Fig. 1c).

**Fig. 2:**
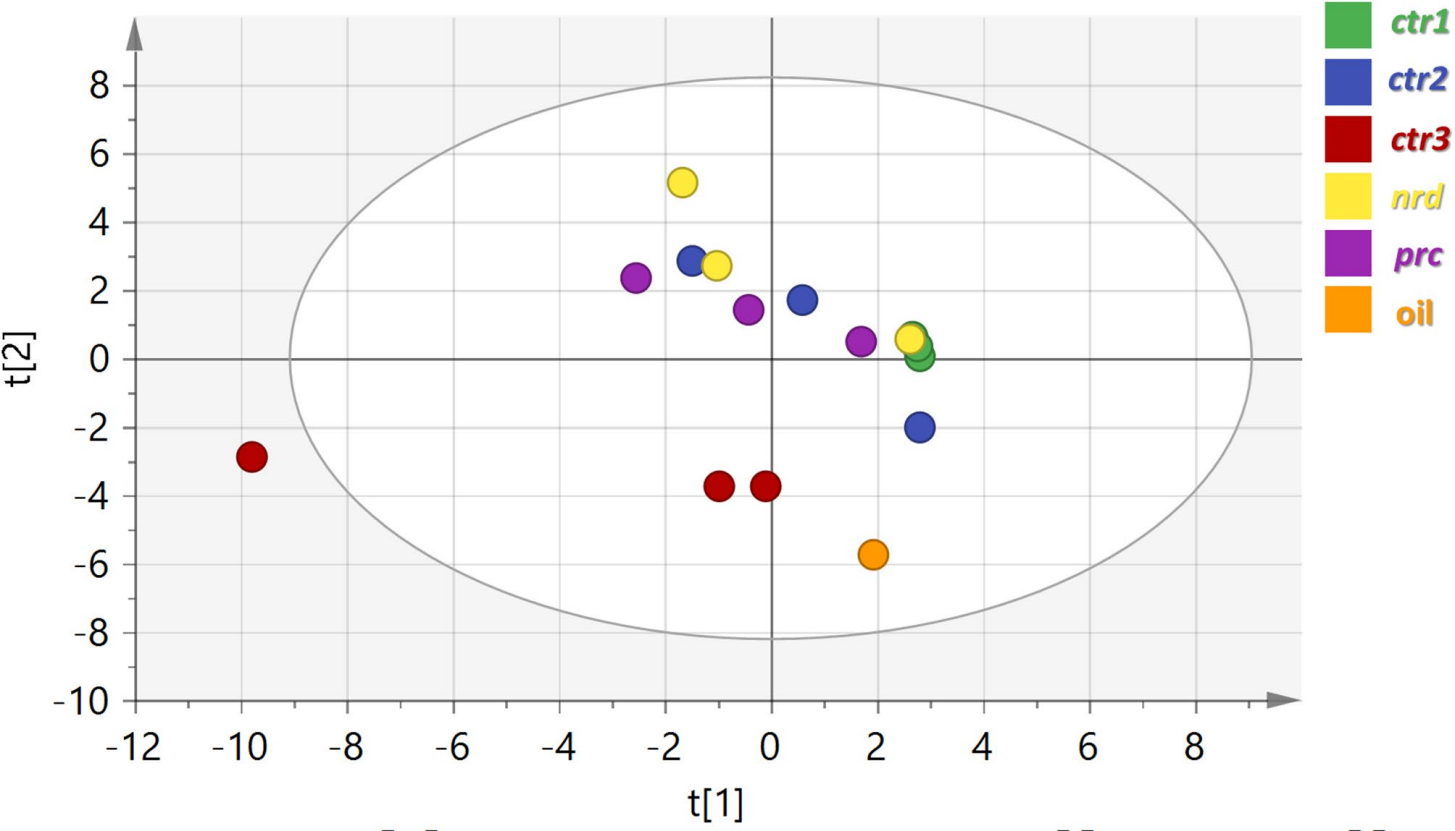
HS-SPME-GC-MS based PCA score plot of PC1 versus PC2 scores of the *Cymbopogon* samples except *C. martinii* (*n* = 3). The sample clusters are placed in two-dimensional space at the distinct locations defined by two vectors of principal component PC1 = 37.91% and PC2 = 68.99%. *ctr1*,* ctr2* and *ctr3*,* C. citratus* leaves from 2 origins; *nrd*,* C. nardus* leaves; *prc*, *C. procerus* leaves; oil, Lemongrass essential oil.

To facilitate visualization of differences among the remaining samples, a second PCA model was constructed after excluding *C. martini*. This model explained 68.99% of the total variance (Fig. 2) and showed partial separation among samples, with the essential oil, *ctr3*, and the remaining samples forming three loosely defined groups. However, the discriminatory capacity of this model was limited with negative prediction power.

**Fig. 3:**
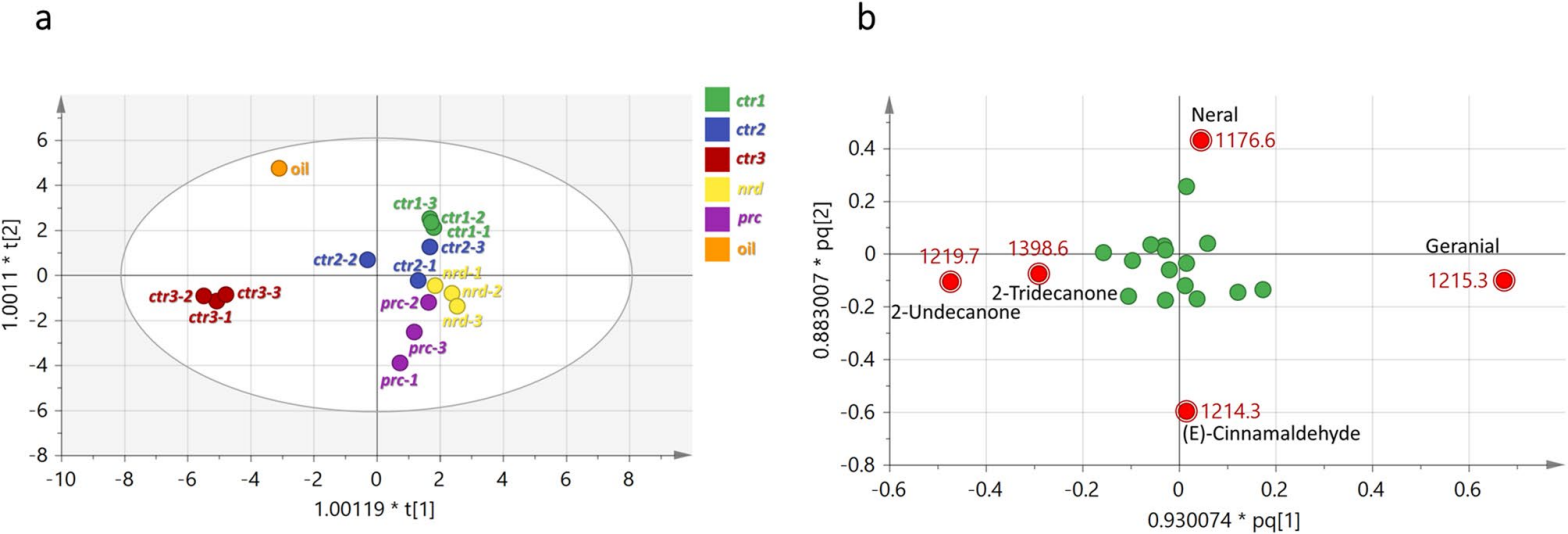
OPLS-DA (**a**) score plot and (**b**) loading plot derived from *Cymbopogon* samples analyzed by GC-MS. The metabolites responsible for the discrimination are shown in the loading plot, colored in red and labeled with their KI values. *ctr1*, *ctr2* and *ctr3*, *C. citratus* leaves from 2 origins; *nrd*, *C. nardus* leaves; *prc*, *C. procerus* leaves; oil, Lemongrass essential oil.

Supervised OPLS-DA was further applied to visualize trends among citral-enriched samples, including *C. citratus* (*ctr1*,* ctr2*,* ctr3*), *C. nardus*,* C. procerus*, and lemongrass essential oil. The model exhibited a cumulative R2Y of 90.28% and a moderate predictive ability (Q2 = 53.55%; Fig. 3). The OPLS-DA score plot (Fig. 3a) suggested partial separation of *ctr3* and the essential oil from other samples along PC1, while separation of *C. procerus* along PC2 was observed. Loading plots (Fig. 3b) indicated higher contributions of (E)-cinnamaldehyde in *C. procerus* and fatty acyl ketones, such as 2-undecanone and 2-tridecanone, in *ctr3*. These features further supported statistical results presented in the previous section.

Permutation testing and CV-ANOVA results are provided in **Supplementary Fig. ****S2** to assess model robustness, with intercept of negative Q2 value and p-value < 0.05 verifying model validity.

### Screening the vapor-phase antibacterial activity of Cymbopogon leaves, essential oil, and citral

Increasing evidence supports the antimicrobial efficacy of essential oils in the vapor phase, with vapor-phase assays offering advantages over direct-contact methods that may influence antimicrobial outcomes^30,68^. Despite growing interest in vapor-phase antimicrobial activity, the antibacterial effect of volatiles emitted directly from *Cymbopogon* leaves against resistant Gram-positive and Gram-negative pathogens has not been previously reported.

**Fig. 4:**
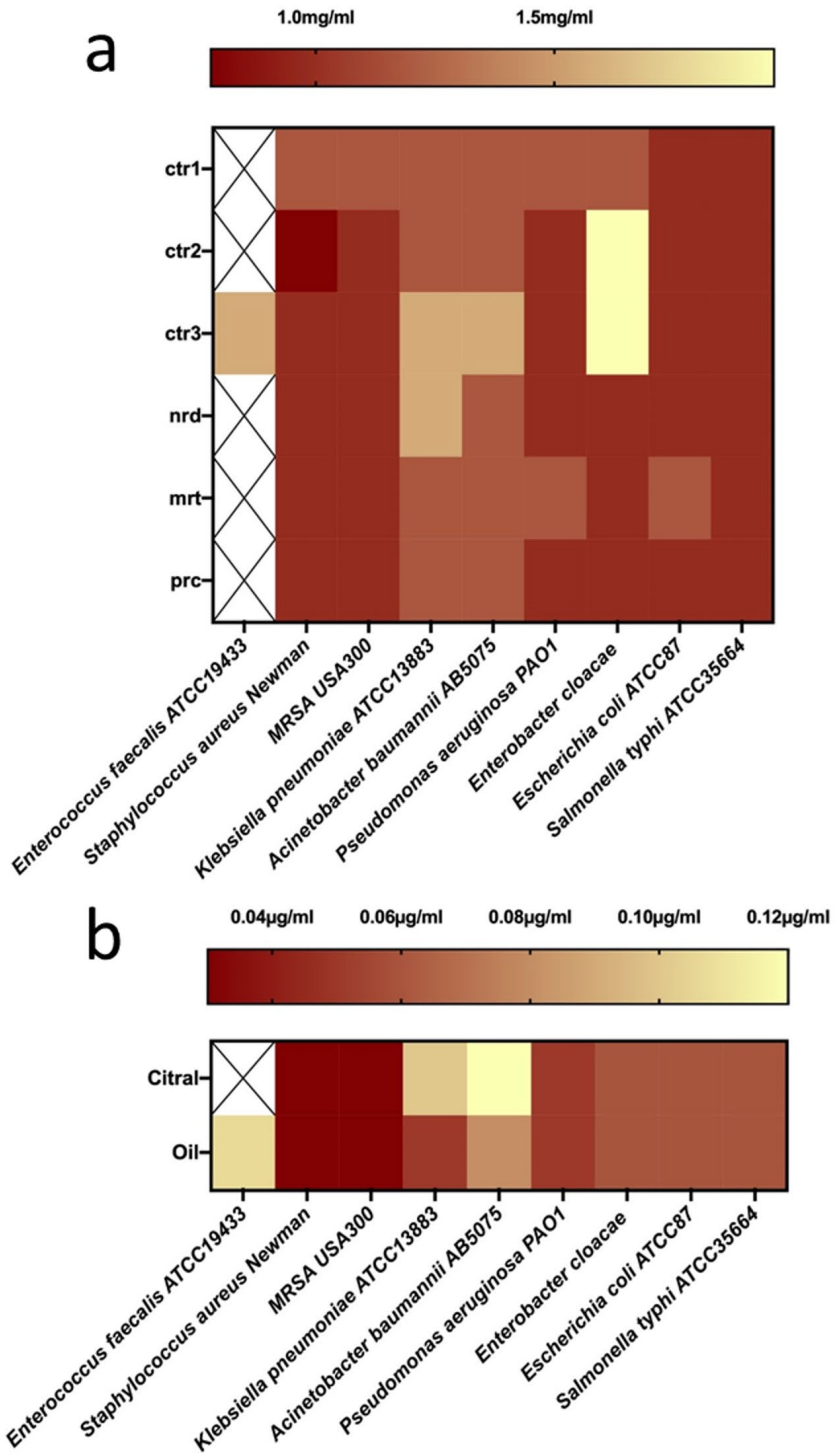
Heatmap showing the vapor-phase antibacterial activity of the tested leaves (*n* = 3), (**a**) and citral and lemongrass essential oil (**b**). The antibacterial activity is represented by mean of vapor-phase minimum inhibitory concentration (VP-MIC). Dark red and yellow represent the lowest and highest VP-MIC (mg/mL or µg/mL), respectively. Symbol “X” means that there was no antibacterial activity detected within the tested concentrations.

The vapor phase of leaves from *C. citratus* (*ctr1*, *ctr2*, *ctr3*), *C. nardus* (*nrd*), *C. martini* (*mrt*), and *C. procerus* (*prc*), as well as lemongrass essential oil and the major compound citral, exhibited measurable antibacterial activity against the tested bacterial resistant strains **(**Table 2; Fig. 4**and Fig. S3**). Variations in VP-MIC values were observed among samples with different dominant constituents. For example, samples dominated by citral (*ctr1*, *ctr2*, *ctr3*, *nrd*, *prc* and lemongrass essential oil) showed lower VP-MIC values (non-significant at *p* < 0.05) against *E. coli* compared to the geraniol-dominant *C. martini* sample (VP-MIC 0.98 mg/mL vs. 1.18 mg/mL, respectively). These observations are consistent with previous reports indicating that aldehydes often exhibit stronger antibacterial activity than alcohols^20,22^.

All *Cymbopogon* samples demonstrated antibacterial activity against most tested ESKAPE pathogens, with the exception of *Enterococcus faecalis*. Among the leaf samples, *ctr3* was the only specimen that exhibited significant antimicrobial activity (at *p* < 0.05) against *E. faecalis* ATCC19433 (VP-MIC 1.57 mg/mL; Fig. 4, **Fig. S3**).

Importantly, lemongrass essential oil exhibited significantly lower VP-MIC values than citral against *E. faecalis* (0.1 µg/mL vs. no detectable activity) (Fig. 4). Notably, although citral represents the major constituent, its isolated form showed weaker or no activity compared with the whole *ctr3* and essential oil. This suggests that the observed antibacterial effect is likely influenced by the combined action of multiple volatile constituents rather than by a single compound, and warrants further targeted assays.

Statistical analysis of VP-MIC values using two-way ANOVA followed by Šídák’s post hoc test revealed significant effects of both sample type and bacterial strain (*p* < 0.05; **Supplementary Table ****S2**). In particular, *ctr3* differed significantly from *ctr1* and *ctr2* against *E. faecalis*, while significant differences among selected leaf samples were also observed for Enterobacter cloacae Table 3.

**Table 3:** Vapor-phase antibacterial activity of the tested leaves, essential oil and citral.

Sample	Vapor-phase minimum inhibitory concentration (VP-MIC)#								
	Enterococcus faecalis ATCC19433	Staphylococcus aureus Newman	MRSA USA300	Klebsiella pneumoniae ATCC13883	Acinetobacter baumannii AB5075	Pseudomonas aeruginosa PAO1	Enterobacter cloacae	Escherichia coli ATCC8739	Salmonella typhi ATCC35664
***ctr1***	*	1.18 ± 0	1.18 ± 0^a^	1.18 ± 0	1.18 ± 0	1.18 ± 0	1.18 ± 0	0.98 ± 0.34	0.98 ± 0.34
***ctr2***	*	0.78 ± 0.34	0.98 ± 0.34	1.18 ± 0	1.18 ± 0	0.98 ± 0.34	1.96 ± 0.68	0.98 ± 0.34	0.98 ± 0.34
***ctr3***	1.57 ± 0.68	0.98 ± 0.34	0.98 ± 0.34	1.57 ± 0.68	1.57 ± 0.68	0.98 ± 0.34	1.96 ± 0.68	0.98 ± 0.34	0.98 ± 0.34
***nrd***	*	0.98 ± 0.34	0.98 ± 0.34	1.57 ± 0.68	1.18 ± 0	0.98 ± 0.34	0.98 ± 0.34	0.98 ± 0.34	0.98 ± 0.34
***mrt***	*	0.98 ± 0.34	0.98 ± 0.34	1.18 ± 0	1.18 ± 0	1.18 ± 0	0.98 ± 0.34	1.18 ± 0	0.98 ± 0.34
***prc***	*	0.98 ± 0.34	0.98 ± 0.34	1.18 ± 0	1.18 ± 0	0.98 ± 0.34	0.98 ± 0.34	0.98 ± 0.34	0.98 ± 0.34
**Essential oil**	0.1 ± 0.03	0.03 ± 0	0.03 ± 0	0.05 ± 0.02	0.08 ± 0.03	0.05 ± 0.02	0.06 ± 0	0.06 ± 0	0.06 ± 0
**Citral**	*	0.03 ± 0	0.03 ± 0	0.10 ± 0.03	0.12 ± 0	0.05 ± 0.02	0.06 ± 0	0.06 ± 0	0.06 ± 0

**#** VP-MIC is expressed as mean ± std (mg/mL for plant leaves and µg/mL for essential oil and citral), *n* = 3. Different concentration units were used to reflect the substantial differences in volatility and effective vapor-phase concentrations among sample types.

* No antibacterial activity was detected against the tested organism.

## Conclusion

This study provides a comparative characterization of volatile profiles emitted from leaves of 4 *Cymbopogon* species using headspace SPME–GC–MS, together with evaluation of a commercial lemongrass essential oil and assessment of vapor-phase antibacterial activity. A total of 44 volatile compounds were identified, with aldehydes-mainly citral isomers (neral and geranial)-predominating in *C. citratus*, *C. nardus*, *C. procerus*, and lemongrass essential oil, whereas *C. martini* was characterized by a high abundance of geraniol. Multivariate data analysis indicated limited discrimination of samples according to geographical origin under the present experimental design.

Vapor-phase antibacterial assays demonstrated inhibitory activity of leaf-emitted volatiles, essential oil, and citral against most tested multidrug-resistant ESKAPE pathogens. Notably, only *C. citratus* (*ctr3*) and lemongrass essential oil exhibited significant inhibitory activity against *Enterococcus faecalis*, while the essential oil exhibited non-significant lower VP-MIC values than citral against *Klebsiella pneumoniae* and *Acinetobacter baumannii.* This observation suggests that antibacterial activity is likely influenced by the overall volatile composition rather than by citral alone, highlighting the potential role of minor constituents.

To the best of our knowledge, this study represents the first combined report on the volatile profile and vapor-phase antibacterial activity of *C. procerus* leaves.

Our future perspective will focus on assessment of the major detected volatiles separately regarding their Vapor phase antimicrobial properties. Overall, the findings highlight the potential of *Cymbopogon* leaf volatiles as natural sources of antimicrobial agents and provide a basis for future studies employing expanded sampling and targeted analyses to further elucidate structure–activity relationships and practical applications. Furthermore, *Cymbopogon* leaf volatiles represent a promising natural source of antimicrobial agents with potential applications in controlling resistant ESKAPE pathogens in hospital environments, food storage, and packaging systems.

## Supplementary Information

Below is the link to the electronic supplementary material.

Supplementary Material 1

Supplementary Material 2

## Data Availability

Data is provided within the manuscript or supplementary information files.
