# Supplementary material for: Headspace GC–MS volatiles profiling in leaves of 4 Cymbopogon species and their vapor-phase antibacterial effects
Source: Sci Rep. 2026 Apr 17;16:12718. doi: 10.1038/s41598-026-45553-7 (PMC13090375; doi:10.1038/s41598-026-45553-7)
Supplement: Supplementary file 2 — Supplementary Material 2 [file 41598_2026_45553_MOESM2_ESM.docx]

**Comparative Headspace GC–MS Profiling of Volatiles from Leaves of Four *Cymbopogon* Species and Their Vapor-Phase Antibacterial Effects**

**Mariam O. Wahdan^1,5^, Fatema R. Saber^2^, Mariam Hassan^3,4^, Marwa E. Hassan^5^, Mohamed A. Farag^2*^**

^1^ Postgraduate Program in Pharmacognosy Department, Faculty of Pharmacy, Cairo University, Kasr El Aini St., Cairo, 11562, Egypt

^2^ Pharmacognosy Department, Faculty of Pharmacy, Cairo University, Kasr El Aini St., Cairo, 11562, Egypt

^3^ Department of Microbiology and Immunology, Faculty of Pharmacy, Cairo University, Kasr El Aini St., Cairo 11562, Egypt

^4^ Department of Microbiology and Immunology, Faculty of Pharmacy, Galala University, New Galala City, Suez, Egypt

^5^ Egyptian Drug Authority, Giza, 12553, Egypt

*Corresponding authors

**Assoc. Prof. Fatema R. Saber**, Email: Fatema.saber @pharma.cu.edu.eg, **Prof. Dr. Mohamed A. Farag, Ph.D.**

Email: [mohamed.farag@pharma.cu.edu.eg](mailto:mohamed.farag@pharma.cu.edu.eg); Tel.: 01004142567


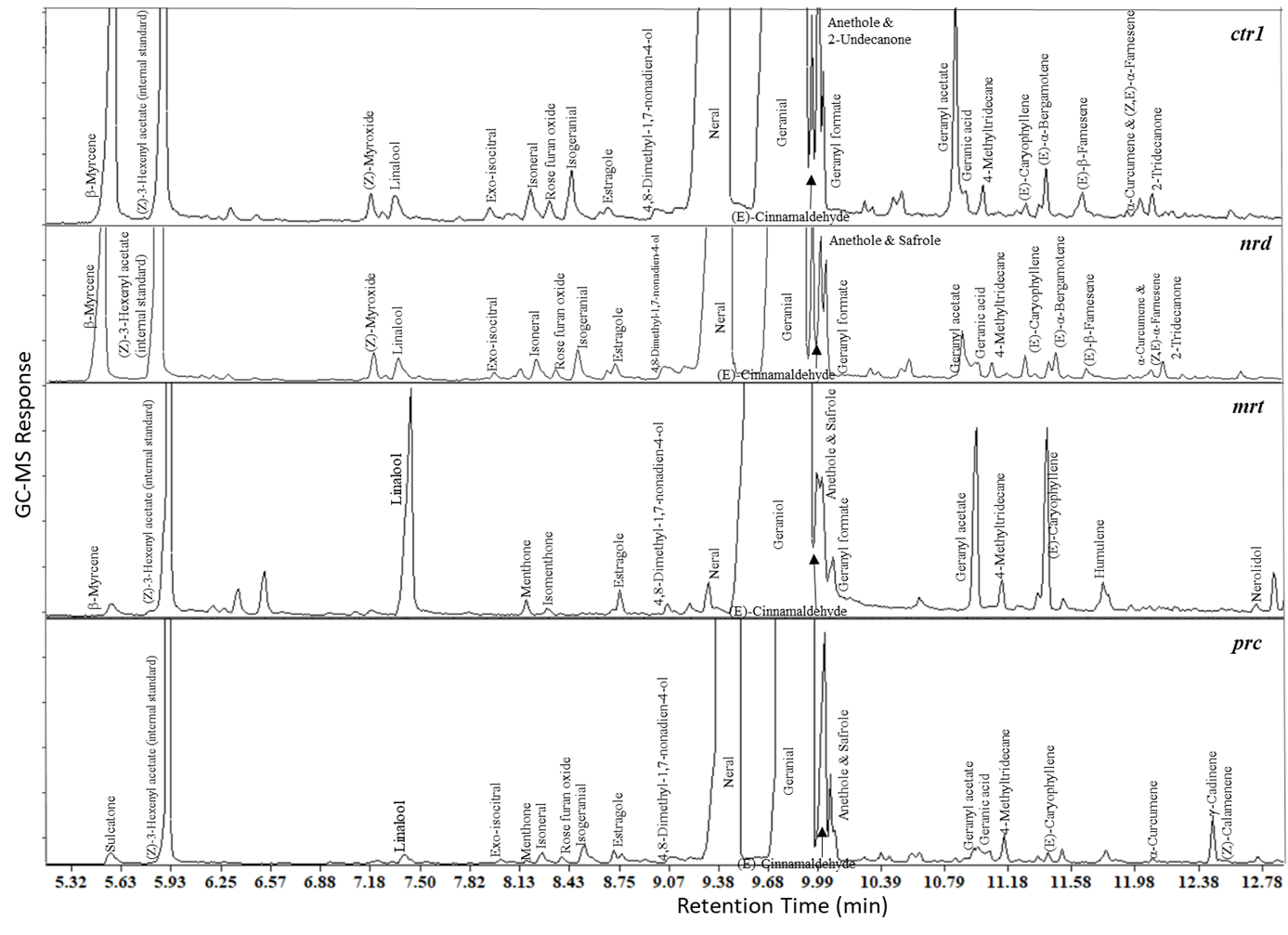


**Supplementary Figure S1:** Representative GC–MS chromatogram of *C. citratus* (*ctr1*), *C. nardus* (*nrd*), *C. martini* (*mrt*) and *C. procerus* (*prc*) leaves’ aroma.


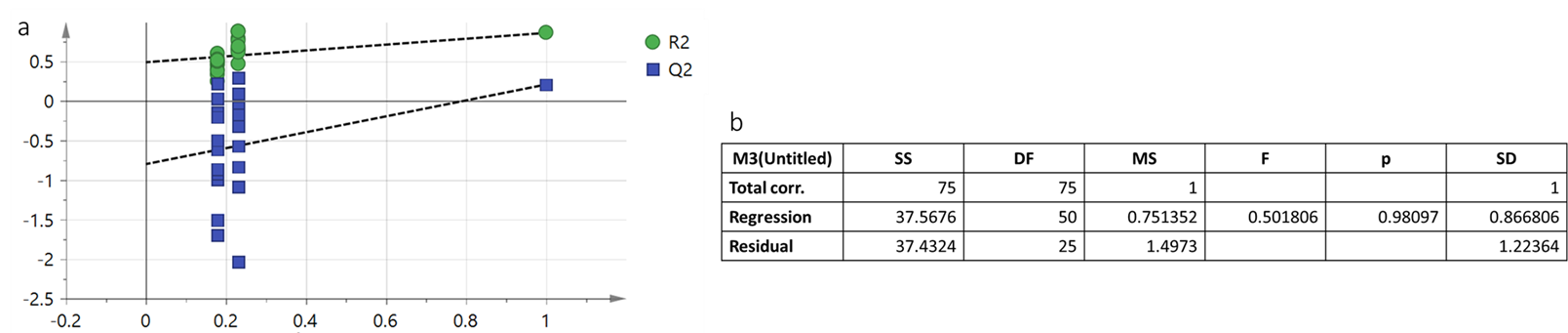


**Supplementary Figure S2:** OPLS-DA optimization and validation parameters for modeling *Cymbopogon* samples. **a.** Permutation test, *n* = 100 that showed negative Q2 intercept value. **b.** CV-ANOVA to assess for model statistical significance showed p value of 0.98097


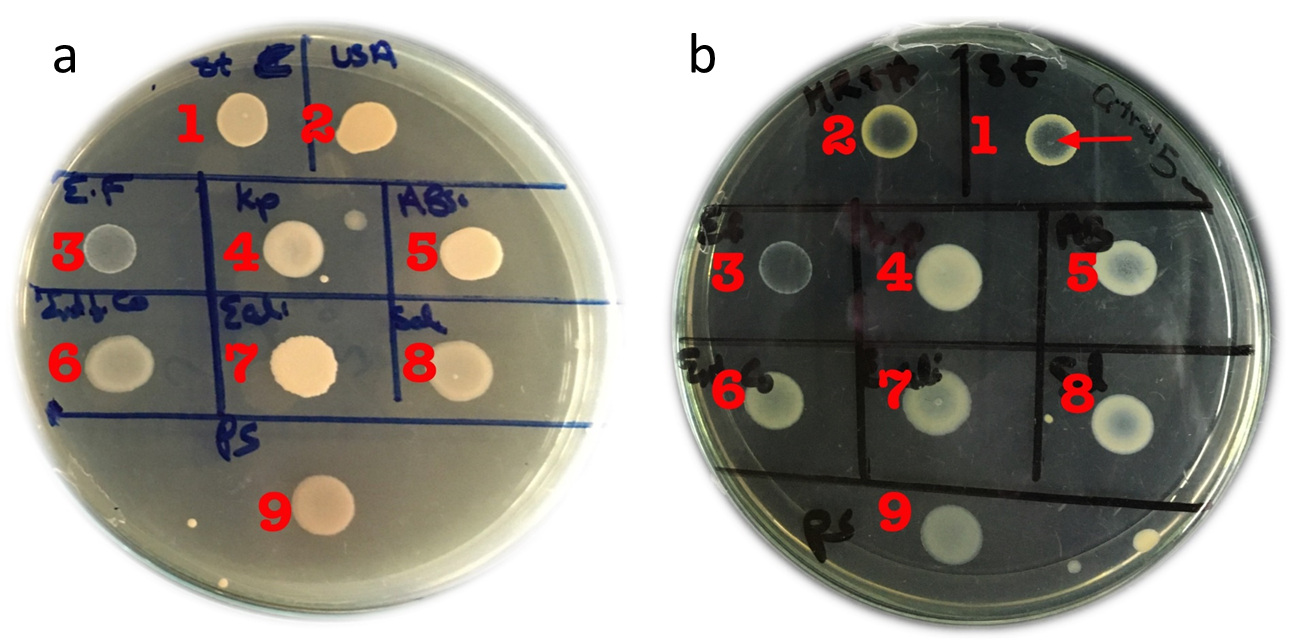


**Supplementary Figure S3**: Vapor-phase minimum inhibitory concentration assay (VP-MIC). (**a)** Control plate showing bacterial growth of the nine tested microorganisms. (**b)** Sample plate showing inhibition of bacterial growth. The arrow in panel (**b)** indicates growth suppression of the tested microorganism. The numbers 1-9 on panels (**a)** and (**b)** correspond to the tested microorganisms as follows: 1, *Staphylococcus aureus* Newman; 2, methicillin-resistant *Staphylococcus aureus* (MRSA USA300); 3, *Enterococcus faecalis* ATCC19433; 4, *Klebsiella pneumoniae* ATCC13883; 5, *Acinetobacter baumannii* AB5075; 6, *Enterobacter cloacae*; 7, *Escherichia coli* ATCC87; 8, *Salmonella typhi* ATCC35664; and 9, *Pseudomonas aeruginosa* PAO1. All nine strains were tested simultaneously on the same plate under identical headspace and incubation conditions. The VP-MIC was defined as the lowest concentration of the tested leaf sample, essential oil, or citral causing visual growth suppression relative to the control.
